# Invertible micellar polymer nanoassemblies target bone tumor cells but not normal osteoblast cells

**DOI:** 10.4155/fso.15.14

**Published:** 2015-11-01

**Authors:** Olena Kudina, Kristen L Shogren, Carl T Gustafson, Michael J Yaszemski, Avudaiappan Maran, Andriy Voronov

**Affiliations:** 1 Department of Coatings & Polymeric Materials, North Dakota State University, Fargo, ND 58105-6050, USA (currently Department of Science & Technology University of Twente, the Netherlands); 2 Department of Orthopedics, Mayo Clinic, Rochester, MN 55905, USA; 3 Department of Coatings & Polymeric Materials, North Dakota State University, Fargo, ND 58105-6050, USA

**Keywords:** bone tumor treatment, curcumin delivery, invertible polymer micelles, micellar nanoassemblies

## Abstract

**Aim:**

To demonstrate the capability of the invertible micellar polymer nanoassemblies (IMAs) to deliver and release curcumin using the recently discovered mechanism of macromolecular inversion to treat bone tumor cells.

**Materials & Methods:**

The effect of IMA-mediated delivery of curcumin on osteosarcoma cell survival was investigated using MTS assays. To assess the effect of IMAs-delivered curcumin on osteosarcoma cell growth, fluorescence-activated cell sorting was performed. The uptake of micellar nanoassemblies was followed using confocal microscopy.

**Results & Discussion:**

IMAs-delivered curcumin is effective in blocking osteosarcoma cell growth. It decreases cell viability in human osteosarcoma (MG63, KHOS, and LM7) cells while having no effect on normal human osteoblast cells. It indicates that curcumin-loaded IMAs provide a unique delivery system targeted to osteosarcoma cells.

## Abstract (lay abstract)

Osteosarcoma is the most common primary bone malignancy that predominantly affects children and adolescents. Curcumin, a principal substance in the Asian spice turmeric, has been shown to block osteosarcoma cell growth. The clinical development of curcumin has been hindered due to poor aqueous solubility and thus, bioavailability, restricting its use as a drug. In this study, in order to improve the bioavailability and efficacy of curcumin, the drug was loaded (solubilized) into invertible micellar polymer nanoassemblies made from amphiphilic invertible polymers.

**Figure 1.**
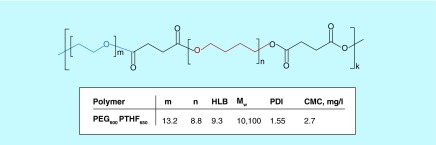
**Chemical structure and characteristics of the PEG600PTHF650 used in the study.** The chemical structure of the PEG_600_PTHF_650_ used in this study, and the molecular weight, polydispersity index, hydrophilic lipophilic balance and critical micelle concentration of the polymer sample**.** The chemical structure of the PEG_600_PTHF_650_ was confirmed by proton nuclear magnetic resonance (^1^H NMR) spectroscopy and Fourier transform infrared spectroscopy [28]. To confirm the formation of micelles from PEG_600_PTHF_650_ in aqueous solution, CMC was measured via solubilization of a fluorescent probe, pyrene, to study the association behavior of amphiphilic polymers [28]. CMC: Critical micelle concentration; HLB: Hydrophilic lipophilic balance; m: Number of PEG fragments in amphiphilic invertible polymers macromolecules; Mw: Weight average molecular weight; n: Number of PTHF fragments in amphiphilic invertible polymers macromolecules; PDI: Polydispersity index.

**Figure 2.**
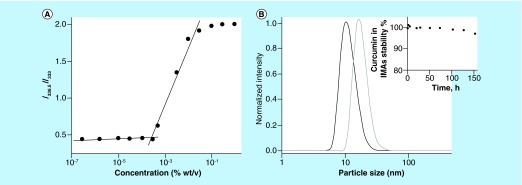
**Physico-chemical properties of invertible micellar polymer nanoassemblies.** **(A)** The intensity ratio (*I_336.5_*/*I_333_*) of the excitation spectra of pyrene in PEG_600_PTHF_650_ solutions versus polymer concentration. **(B)** The sizes of blank and curcumin-loaded polymer micellar nanoassemblies as determined by dynamic light scattering. IMA: Invertible micellar polymer nanoassembly.

**Figure 3.**
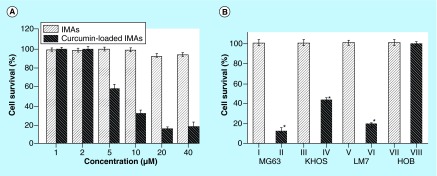
**Osteosarcoma cell survival.** MG63 osteosarcoma cells were treated with IMAs and IMA-loaded curcumin at different concentrations for 48h **(A)**. Effect of curcumin delivery on MG63 KHOS, LM7 and HOB cells at 72 h **(B)**. IMAs: I, III, V, VII; curcumin-loaded IMAs: II, IV, VI, VIII. HOB: Human osteoblast; IMA: Invertible micellar polymer nanoassembly.

**Figure 4.**
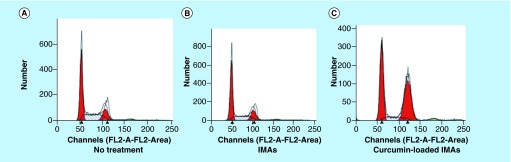
**Effect of invertible micellar polymer nanoassemblies-delivered curcumin on cell cycle.** MG63 osteosarcoma cells were analyzed by fluorescence-activated cell sorting analysis after 24 h: **(A)** untreated, **(B)** IMAs and **(C)** curcumin-loaded IMAs. IMA: Invertible micellar polymer nanoassembly.

**Figure 5.**
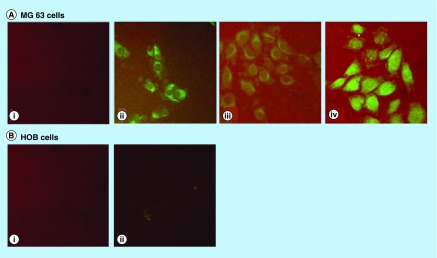
IMA-delivered curcumin uptake in bone cells; 20 μM IMAs (I) and IMA-loaded curcumin (II–IV) were exposed to MG63 osteosarcoma cells and normal human osteoblasts (HOB) for 30 min (I, II); 1 h (III) and 2 h (IV). No fluorescence was observed in HOB at 1 and 2 h. HOB: Human osteoblast; IMA: Invertible micellar polymer nanoassembly.

Osteosarcoma is the most common type of malignant bone tumor in children and young adults, and it is the sixth leading cancer in children under 15 years of age. A second peak in incidence that occurs in the elderly is usually associated with underlying bone pathology, such as Paget's disease, medullary infarct or prior irradiation [1,2]. The prognosis for osteosarcoma is often poor, as 30% of patients with that diagnosis eventually develop lung metastases [1,3,4]. The standard clinical treatment consists of presurgical (neoadjuvant) chemotherapy, followed by surgical resection of the primary tumor after the second or third cycle of the year-long chemotherapy regimen. Cisplatin, doxorubicin, adriamycin, methotrexate, ifosfamide and etoposide are the drugs most commonly used in the treatment of osteosarcoma [5,6].

Curcumin, which is the principle curcuminoid constituent of the spice turmeric, has been investigated for its therapeutic effects on several cell types, including osteosarcoma and other cancers [7–13]. Several signaling mechanisms have been suggested for the action of curcumin in osteosarcoma and other cell types, and the mechanism appears to be cell type specific. However, the therapeutic dose and the intracellular uptake are not fully known yet. Although curcumin has not been shown to have any side effects in clinical trials, its clinical application is currently limited due to its low bioavailability and poor solubility in aqueous medium (11 ng/ml) and its low bioavailability. Even after high amounts of oral administration (8 g/day), extremely low levels of curcumin (22–41 ng/ml) have been reported in humans [14,15]. One possible way to enhance curcumin aqueous solubility and bioavailability is to conjugate its molecules with polymers [16,17].

There is firm evidence that long-circulating polymeric micelles can accumulate effectively in tumors, due to microvascular permeability to circulating macromolecules and their lymphatic drainage in solid tumors [18–20]. Polymeric micelles (self-assemblies) are species with hydrophilic exteriors and hydrophobic interiors that provide an environment that accommodates water-insoluble molecules, including curcumin. The size of these assemblies, typically 10–100 nm, can be controlled by varying the length of the hydrophobic fragment in the amphiphilic polymers [18,21].

Amphiphilic invertible polymers (AIPs), which were synthesized by our group, can be considered to be promising polymer candidates for micellar delivery of lipophilic (poorly water-soluble) drugs. In previous studies, we demonstrated that AIP macromolecules form invertible micellar nanoassemblies (IMAs) by increasing polymer concentration, solubilizing lipophilic drugs in the interiors of IMAs and changing the macromolecular conformation by making local changes to the environmental polarity [22–27]. The latter unique ability can be a decisive factor in AIP-mediated controlled delivery using a new and different inversion mechanism of lipophilic drugs release to various cancerous cells. In our most recent work, curcumin-loaded IMAs displayed anticancer efficiency by delivering water-insoluble drugs to breast carcinoma cells [28]. In addition, the loading into micellar polymer nanoassemblies significantly improved the bioavailability of curcumin in aqueous medium. As a result, it was shown that, being nontoxic against human cells, IMAs are able to solubilize, deliver and release poorly water-soluble curcumin molecules to treat tumor cells [28]. Depending on the chemical structure of the AIP, the release of curcumin from IMAs might result exclusively from macromolecular inversion due to changes in local environmental polarity. The release profile can be controlled by AIP structure and concentration. The curcumin-loaded micellar polymer nanoassemblies were stable in a homogenous polar aqueous medium, but once they approach the less polar cell biomembrane surface, stimuli-triggered conformational inversion occurred [28]. The latter enhances interaction between the drug and the biomembrane, and thus, drug release from the nanoassemblies.

Such behavior by certain AIP formulations demonstrates the potential of IMA-based responsive polymer micellar vehicles as a promising platform for controlled delivery of poorly water-soluble drug candidates by means of new stimuli-responsive release mechanisms.

## Materials & methods

### Polymer synthesis, characterization & formation of micellar nanoassemblies

Poly(ethylene glycol) (PEG, molecular weight 600 g/mol), polytetrahydrofuran (PTHF, molecular weight 650 g/mol), pyrene, carbon tetrachloride (ACS reagent, 99.9%), methanol (ACS reagent, 99.8%) and deuterium oxide (99.9 atom% D) were purchased from Sigma-Aldrich (St.Louis, MO, USA) and used as received.

The polymer used in this study was synthesized from PEG-600 and PTHF-650 (PEG_600_PTHF_650_) using previously reported methods [28,29]. Critical micelle concentration (CMC) of the polymer was measured with a pyrene fluorescent probe, using a previously reported method to measure solubilization [28]. The spectra were taken using a FluoroMax-3 fluorescence spectrometer (Jobin Yvon-Horiba, Kyoto, Japan) with 90° geometry and a slit opening of 0.5 nm. For fluorescence excitation spectra, λ_em_ = 390 nm was chosen. The spectra were accumulated with an integration time of 0.5 nm/s. CMC values were determined after fitting the semilogarithmic plots of intensity ratio *I_336.5_/I_333_* versus log concentration to the sigmoidal curve. The size distribution and zeta potential of the AIP micelles were measured using a Malvern (Worcestershire, UK) Zetasizer Nano ZS90 at 25 °C. The final numbers represent an average of a minimum of five (size) or ten (zeta potential) individual measurements. Proton nuclear magnetic resonance spectra were recorded on a JEOL ECA 400MHz NMR spectrometer using chloroform-*d* and deuterium oxide as solvents.

### Study on interactions of IMAs with osteosarcoma cells

#### Cell culture

Human osteosarcoma (MG63, KHOS and LM7) and primary human osteoblast (HOB) cells were plated in 24 well culture plates (25,000 cells/cm^2^) in 1 ml of medium (D2906 Dulbecco's Modified Eagle's Medium/Ham's F-12 Nutrient Mixture F-12 Ham; Sigma) supplemented with 10% charcoal stripped fetal calf serum (Thermo Fisher Scientific, Logan, UT, USA), 0.9 M sodium bicarbonate, 10 U/ml penicillin G and 100 µg/ml streptomycin (Invitrogen, Carlsbad, CA, USA). Cells were maintained at 37 °C in 5% CO_2_.

#### Biological activity of released curcumin

To determine the biological activity of curcumin released from polymers, Transwells (Corning, NY, USA) containing OPF or OPF/PLGA composites, with or without curcumin, were transferred to the wells with osteosarcoma cells in culture 24 h after plating the cells. At the end of three days, an MTS (3-(4,5-dimethylthiazol-2-yl)-5-(3-carboxymethoxyphenyl)-2-(4-sulfophenyl)-2H-tetrazolium) colorimetric assay (Promega Corporation, Madison, WI, USA) was used to assess cell survival.

#### Cell cycle distribution in micellar curcumin-treated cells

The cell cycle analysis was carried out as described previously [30]. Briefly, MG63 osteosarcoma cells (1 × 10^6^ cells) were placed in T-75 flasks and were untreated or treated with 20 μM polymer or polymer containing curcumin. After 24 h of treatment, the cells were trypsinized, spun and washed with phosphate buffered saline. Each sample was fixed on ice by adding 300 µL of cold 95% ethanol, dropwise, for 5 min. The cells were then fixed on ice for 60 min, then washed three times with phosphate buffered saline and resuspended in RNase A solution. The fixed cells were stained with propidium iodide (50 µg/ml) and analyzed using a fluorescence-activated cell sorting scan unit (Becton, Dickinson and Company, San Jose, CA, USA). The relative proportions of cells in the G1, S and G2/M cell cycle phases were estimated by compartment analysis of DNA fluorescence using software from the manufacturer.

#### Curcumin uptake

MG63 osteosarcoma and HOB cells were incubated with 20 mM polymer or miciller curcumin for 30, 60 and 120 min and examined by confocal microscopy.

#### Statistical methods

All data are reported as mean ± standard error for n = 3; the data are representative of three independent experiments. One-way ANOVA was used to detect statistically significant differences between groups. P values <0.05 were considered significant.

## Results & discussion

In this study, to demonstrate the capability of the most promising IMA formulations to deliver and release curcumin using the macromolecular inversion mechanism to treat bone tumor cells, drug-loaded IMAs were applied to different osteosarcoma cell lines.

### AIP synthesis & self-assembly

As shown in our previous work, curcumin-loaded IMAs based on AIP from poly(ethylene glycol) (PEG, molecular weight 600 g/mol) and polytetrahydrofuran (PTHF, 650 g/mol) (PEG_600_PTHF_650_) are stable in aqueous environments [28]. However, AIP macromolecules undergo rapid inversion of macromolecular conformation, due to subtle changes in the polarity of the environment [24,27]. Once the curcumin-loaded IMAs from PEG_600_PTHF_650_ reach the cell surface sites (less polar than aqueous medium), drug release is induced by the conformational changes of the AIP macromolecules [28].

In this study, we used PEG_600_PTHF_650_ micellar nanoassemblies for curcumin delivery and release, using a recently discovered inversion mechanism to target different osteosarcoma cell lines, and showed how this mechanism can be used for specific tumor cell treatment using poorly water-soluble drugs.

Figure 1 shows the chemical structure of the PEG600PTHF650 used in this study, as well as the molecular weight, polydispersity index, hydrophilic lipophilic balance and critical micelle concentration of the polymer sample.

Pyrene excitation spectra were monitored in the wavelength range of 300–360 nm. A redshift in the fluorescence excitation spectra from 333 to 336.5 nm with an increasing polymer concentration in aqueous solution indicates solubilization of pyrene within the micellar hydrophobic environment, as well as the transfer of pyrene molecules from water to the polymer micelles. The sharp increase in the intensity ratio corresponds to the CMC of PEG_600_PTHF_650_ in aqueous solution (Figure 2A).

As demonstrated in our previous work, increasing the concentration of PEG_600_PTHF_650_ in water leads to the formation of micellar nanoassemblies due to the aggregation of single unimolecular micelles and the formation of hydrophilic and lipophilic domains [28,31]. Formation of the polymer micellar nanoassemblies was confirmed by the characteristic ^1^H NMR spectra of PEG_600_PTHF_650_ taken in deuterium oxide over the wide polymer concentration range.

### Characterization of curcumin-loaded IMAs

To carry out the physicochemical characterization of curcumin-loaded micelles, the drug was incorporated into the IMAs using 1% polymer solutions. At this concentration, the micelles are self-assembled into the IMAs; the hydrophobic curcumin was solubilized through physical interactions with the polymer hydrophobic fragments of the interiors of the IMAs. Ultraviolet-visible (UV-vis) spectroscopy measurements were performed to determine loading. Table 1 shows the curcumin loading content in micellar formulation. Figure 2B shows the average hydrodynamic micellar diameter and size distribution before and after drug loading in the micellar nanoassemblies. When micelles are loaded, the diameters of the IMAs are larger, thus confirming drug incorporation into the micellar interior.

The polymer micellar nanoassemblies are small, with narrow size distribution, which are physical properties conducive for use as nanocarriers for drug delivery. Zeta potential analysis of the micellar nanoassemblies before and after curcumin loading shows a negative surface charge of the micellar nanoassemblies at room temperature (Table 1) that decreases but remains negative after incorporating curcumin into the IMA's interior.

It is well known that curcumin is not stable at neutral and basic pH and decomposes at about 90% within 30 min of placement in phosphate-buffered saline at pH 7.2. In this study, the chemical stability of curcumin in IMAs was monitored using UV-vis spectroscopy. The recorded data show that the change in absorbance of curcumin-loaded IMAs in time is small for micellar polymer nanoassemblies based on PEG_600_PTHF_650_. It confirms the very good stability of micellar curcumin and indicates that drug bioavailability can be enhanced significantly by incorporating curcumin into IMAs (Figure 2B, inset).

The overall results demonstrate that a high amount of curcumin can be incorporated into the IMAs from PEG_600_PTHF_650_, and that the physicochemical properties of loaded IMAs are promising for testing micelle-mediated drug delivery and curcumin uptake in osteosarcoma cells.

### Effect of IMA-mediated curcumin delivery in osteosarcoma cells

The effect of IMA-mediated delivery of curcumin on osteosarcoma cell survival was investigated using MTS assays. The cell survival rate measured at the end of 72 h revealed that curcumin delivered through micellar nanoassemblies decreased survival compared with polymer controls. A dose response study showed that at lower concentrations (1 and 2 μM) both IMA and IMA-loaded curcumin had no effects on cell survival. However, the cell survival rate decreased in the presence of 5, 10, 20 and 40 μM curcumin-loaded IMAs to 61, 33, 20 and 21%, respectively, in MG63 cells (Figure 3A). The effect of nonloaded IMAs was tested at the same concentrations, and cell survival only decreased to 99, 99, 90 and 90% in the presence of 5, 10, 20 and 40 μM, respectively. Our results show that micelle-mediated delivery reduced cell survival in human osteosarcoma cell lines MG63, KHOS and LM7 to 12, 44 and 19%, respectively, at 72 h compared with a polymer control (Figure 3B). IMAs and IMA-delivered curcumin (20 μM) did not affect cell survival in normal primary HOBs (Figure 3B).

The results show that IMA-delivered curcumin kills osteosarcoma (cancer) cells (Figure 3A & B), specifically targeting the osteosarcoma cells and not normal bone cells (Figure 3B). Thus, the observed cytotoxicity of cancer cells is caused by IMAs-mediated curcumin delivery and not by the presence of polymeric micelles. This finding indicates that IMA-based delivery can be considered a powerful approach in the targeted delivery of poorly soluble curcumin in bone cancer cells.

The data show that IMAs effectively deliver curcumin and target three different bone cancer cells (MG63, KHOS and LM7) and not normal HOBs. Previous studies have shown that LM7 is highly metastatic compared with MG63 and KHOS, and that it induces tumors in animals [32]. Further studies are required to understand the clinical relevance of these findings and to examine whether IMAs could be a useful delivery vehicle for treating metastatic osteosarcoma in animal models.

### Effect of IMA-delivered curcumin on growth arrest

To assess the effect of IMAs-delivered curcumin on osteosarcoma cell growth, we performed fluorescence-activated cell sorting as described [30] in the presence of both curcumin-loaded and nonloaded micellar nanoassemblies in MG63 cells. Our results show that curcumin-loaded micelles containing treatment led to a 2.5-fold increase and a corresponding decrease in percentage of cells in G2 phase and S phase (Figure 4C) compared with no treatment, whereas polymeric micelles alone had no effect on cell cycle progression (Figure 4B).

The results confirm earlier reports in the literature that curcumin-mediated cytotoxicity is associated with cell-cycle arrest [33,34]. The data show that IMA-delivered curcumin induces G2 arrest in osteosarcoma cells. Both G1 arrest and G2 arrest contribute to antigrowth and antitumor effects in osteosarcoma cells in the presence of curcumin and other cytotoxic agents. Identifying the genes and signaling pathways that contribute to cell cycle arrest will help in understanding the biological mechanisms associated with targeted curcumin delivery in osteosarcoma cells.

### Uptake of IMA-delivered curcumin in osteosarcoma cells

In order to understand the differential effects of IMA-delivered curcumin in osteosarcoma and normal osteoblast cells, we followed the uptake of micellar nanoassemblies and IMA-loaded curcumin. Cells exposed to IMAs and micellar assembly loaded curcumin were analyzed by confocal microscopy. Our results show that the curcumin was readily taken up in MG63 cells when delivered through micellar nanoassemblies. The drug was taken up by the cytoplasm in 30 min and could still be seen at 2 h (Figure 5A). The confocal microscopy data confirm that that IMAs effectively delivered curcumin and targeted bone cancer cells MG63, KHOS and LM7, but not HOBs (Figure 5B).

A few general conclusions can be made from the experimental results obtained in this study. First, curcumin-loaded IMAs are cytotoxic against three different osteosarcoma cell lines. Successful administration of the drug was demonstrated, and curcumin delivery to all osteosarcoma cells was followed. The results also confirmed that micellar curcumin remains stable for a long period of time under conditions that (if the drug is not solubilized in micellar nanoassemblies) facilitate rapid chemical decomposition of curcumin in water, indicating improved bioavailability of micellar curcumin for bone cancer cellular uptake. Finally, the cytotoxicity results show that curcumin-loaded IMA formulations are highly biocompatible and exhibit no toxicity to HOB cells, thus providing a unique delivery system targeted to osteosarcoma cells. As shown in Figure 3B, the HOB cell survival rate is very high in the presence of both nonloaded IMAs and curcumin-loaded micellar nanoassemblies.

## Conclusion

In summary, our study offers a novel method of polymer-based drug delivery that could be explored further to study the chemopreventive actions of curcumin in osteosarcoma and other cancers. Curcumin is known for its anti-inflammatory and antioxidant properties [35,36] and has been evaluated as a cure in several cancers, based on its low toxicity and high tolerability in patients. However, many patient and animal studies have been unsuccessful in increasing the bioavailability of curcumin; in addition, although curcumin is widely known for its pleiotropic effects and safety, the full therapeutic potential of this compound is not yet understood in many diseases. Further studies are required in order to understand the clinical relevance of these findings and to examine whether IMA could be a useful delivery vehicle for treating metastatic osteosarcoma in animal models.

## Future perspective

Osteosarcoma is the most common primary bone malignancy that predominantly affects children and adolescents. Curcumin, a principal substance in the Asian spice turmeric, has been shown to block osteosarcoma cell growth. The clinical development of curcumin has been hindered due to poor aqueous solubility and thus, bioavailability, restricting its use as a drug. In this study, in order to improve the bioavailability and efficacy of curcumin, the drug was loaded (solubilized) into IMAs made from AIPs.

Strategically designed AIPs are capable of self-assembling into IMAs that can bind with and traverse the membranes of targeted cells, accommodating (solubilizing) curcumin that is otherwise insoluble in water and rapidly inverting their molecular conformation in response to subtle changes in the polarity of the local environment to deliver their cargo across the cell membrane. These specific properties make this unique class of self-assembling macromolecules a comprehensive and potentially attractive system for the development of a new platform for the efficient delivery of curcumin to osteosarcoma cells.

We expect that this efficient inversion mechanism better facilitates the desired carrier–cell interactions and enhances targeted curcumin release. From the combined perspectives of ease of manufacturing and efficient drug delivery, the IMAs we developed are novel and unexplored, with the potential to revolutionize the rapid production of nanoscale osteosarcoma drug delivery vehicles.

**Table 1.** **Physical properties of blank and curcumin-loaded invertible micellar polymer nanoassemblies from PEG_600_ PTHF_650_ (1% w/v).**

**AIP**	**Drug loading (wt.%/wt. polymer)**	**Size (nm; blank)**	**Size (nm; loaded)**	**ζ-potential (mV; blank)**	**ζ-potential (mV; loaded)**
PEG_600_PTHF_650_	10.8 ± 0.3	14.4 ± 0.3	19.9 ± 2.4	-28.2 ± 3.6	-19.4 ± 0.3

AIP: Amphiphilic invertible polymer; wt: Weight.

**Executive summary**

**Aim**

The authors wished to demonstrate the capability of the invertible micellar polymer nanoassemblies (IMAs) to deliver and release curcumin using the recently discovered mechanism of macromolecular inversion to treat osteosarcoma cells.

**Results**

Using UV-vis spectroscopy, it is shown that micellar curcumin remains stable for a long period of time under conditions that facilitate rapid chemical decomposition of curcumin in water, indicating improved bioavailability of micellar curcumin.
Curcumin-loaded IMAs are cytotoxic against three different osteosarcoma cell lines (MG63, KHOS and LM7). Successful administration of the drug and curcumin delivery to all osteosarcoma cells was followed using MTS assays and confocal microscopy (for MG63).
Curcumin-loaded IMA formulations are biocompatible and exhibit no toxicity to human osteoblast cells.
Using fluorescence-activated cell sorting, it is demonstrated that IMA-delivered curcumin induces G2 arrest in osteosarcoma cells.

**Conclusion**

IMAs-delivered curcumin is effective in blocking osteosarcoma cell growth. It decreases cell viability in human osteosarcoma cells while having no effect on normal human osteoblast cells. It indicates that curcumin-loaded IMAs provide a unique delivery system targeted to osteosarcoma cells.
